# Functional Analysis of *GmCPDs* and Investigation of Their Roles in Flowering

**DOI:** 10.1371/journal.pone.0118476

**Published:** 2015-03-03

**Authors:** Miao Wang, Xin Xu, Xinxin Zhang, Shi Sun, Cunxiang Wu, Wensheng Hou, Qingyu Wang, Tianfu Han

**Affiliations:** 1 Ministry of Agriculture Key Laboratory of Soybean Biology (Beijing), Institute of Crop Sciences, the Chinese Academy of Agricultural Sciences, Beijing, 100081, China; 2 College of Plant Science, Jilin University, Changchun, 130062, Jilin, China; Institute of Genetics and Developmental Biology, Chinese Academy of Sciences, CHINA

## Abstract

The onset of floral development is a pivotal switch in the life of soybean. Brassinosteroids (BRs), a group of steroidal phytohormones with essential roles in plant growth and development, are associated with flowering induction. Genes involved in BR biosynthesis have been studied to a great extent in Arabidopsis, but the study of these genes has been limited in soybean. In this study, four CPD homologs (GmCPDs) catalyzing BR synthesis were isolated from soybean. Transcripts were mainly confined to cotyledons and leaves and were down-regulated in response to exogenous BR. Bioinformatic analysis showed strong sequence and structure similarity between GmCPDs and AtCPD as well as CPDs of other species. Overexpression of *GmCPDs* in an Arabidopsis BR-deficient mutant rescued the phenotype by restoring the biosynthesis pathway, revealing the functional roles of each *GmCPDs* in. Except for the rescue of root development, leaf expansion and plant type architecture, *GmCPDs* in expression also complemented the late flowering phenotype of Arabidopsis mutants deficient in CPD. Further evidence in soybean plants is that the expression levels of *GmCPDs* in are under photoperiod control in *Zigongdongdou*, a photoperiod-sensitive variety, and show a sudden peak upon floral meristem initiation. Together with increased *GmCPDs* in expression in the leaves and cotyledons of photoperiod-insensitive early-maturity soybean, it is clear that *GmCPDs* in contribute to flowering development and are essential in the early stages of flowering regulation.

## Introduction

Flowering is one of the most important events in the life cycle of plants, with optimal timing being especially crucial. Therefore, flowering is controlled by numerous interacting endogenous and environmental cues to ensure appropriate conditions for seed production. At least four signaling pathways have been demonstrated to regulate flowering in concert, involving length of day (photoperiodism), winter cold (vernalization), regulation by gibberellins (GAs), and autonomous floral initiation occurring in the absence of any effective environmental signals [1]. In addition, other factors such as ascorbic acid, ethylene, ambient temperature and light quality have been found to be critical in mediating flowering [2–5]. Brassinosteriod is one of these factors currently awaiting study.

Brassinosteroids (BRs) are a class of plant-specific steroid hormones. The effects of BRs span multiple physiological processes and responses, including the induction of cell elongation, xylem differentiation, root development, leaf bending/expansion, senescence and male fertility [6–8]. In Arabidopsis, most BR-deficient mutants, such as the BR-biosynthetic mutants *det2* [9], *dwf4* [10] and *cpd* [11,12], exhibit a delayed flowering time. These mutants over-accumulate different BR precursors as a result of the blocked BR-biosynthetic pathway [10,13], indicating that changes to endogenous BR and BR precursor levels in Arabidopsis affects flowering time. In addition, BRs have been found to modify the flowering induction networks of Arabidopsis by regulating critical flowering-time genes. The BR-insensitive mutant *bri1* was reported to significantly delay flowering of the autonomous-pathway mutant *ld* and *fca* by elevating *FLC* expression [12]. Moreover, *bdbrd1–1*, a BR-biosynthetic mutant from *Brachypodium distachyon*, was found to suppress the expressions of autonomous pathway genes such as *FCA*, *FY*, *FLD*, *FVE* and *LD* [14,15]. Recently, *BZR1*, a positive regulator in the BR signaling, was confirmed to regulate *FLD* expression by directly binding to *FLD* promoter to mediate flowering [16]. All above raise the possibility that BRs promote flowering through autonomous pathway. Another BR related gene, *CONSTITUTIVE PHOTOMORPHOGENESIS AND DWARFISM* (*CPD*), can be regulated by light and may modulate flowering through the photoperiod pathway.

The *CPD* gene was identified from an Arabidopsis T-DNA insertional mutation that causes the constitutive photomorphogenesis and dwarfism (*cpd*) [17]. This gene encodes CYP90A1/ CPD, which belongs to the cytochrome P450 family [17]. CYP90A1/CPD was thought to be an enzyme that catalyzes C-23 hydroxylation because C23-hydroxylated BR precursors but not 22-hydroxylated cathasterone (CT) rescued the *cpd* mutant [17]. However, recent evidence has suggested that the C-23 hydroxylation reaction is catalyzed by CYP90C1 and CYP90D1 [18]. Actually, (22S)-22-hydroxycampesterol (22-OHCR) is a favored substrate of CYP90A1/CPD [19]. It has been demonstrated that CYP90A1/CPD encoded by *CPD* is a C-3 oxidase that is required for the conversion of 22-OHCR to (22S)-22-hydroxycampest-4-en-3-one (22-OH-4-en-3-one). The BR biosynthesis network in Arabidopsis proceeds from campesterol (CR) to brassinolide (BL), which is biologically the most active form of BR [20–22]. The originally proposed BR synthesis route is considered to start at the conversion of CR to campestanol (CN)[22]. However, the latest study has indicated that these steps are non-essential and that a CN-independent BR pathway is the main route based on enzyme substrate preferences [19]. In the CN-independent BR synthesis pathway, CR is first hydroxylated to 22-OHCR, followed by the oxidation to 22-OH-4-en-3-one. Obviously, CYP90A1/CPD encoded by *CPD* catalyzes the early step of the BR biosynthesis pathway, suggesting its pivotal role in BR biosynthesis.

Interestingly, expression of the BR-biosynthetic gene *CPD* was reported to possess diurnal rhythmicity with light regulation superimposed upon circadian control [23]. These transcriptional changes are independent of BR feedback regulation but are accompanied by the diurnal variation of endogenous BR content [23]. Similar to most of the light-responsive genes of GA synthesis, *CPD* is under photoreceptor-specific control mainly through phytochrome signaling, suggesting a mechanism in which light controls physiological functions via BRs [24–27]. The CIRCADIAN CLOCK ASSOCIATED 1 (CCA1) transcription factor has been shown to mediate circadian control and phytochrome-regulated gene expression [28,29]. As a potential binding sequence of CCA1, the AAAATCT motif was therefore speculated to be present in *CPD* promoters [23]. *CCA1* is a major gene involved in the circadian clock. In Arabidopsis, the circadian clock has dramatic effects on flowering time through the CO–FT photoperiodic flowering pathway modulated by its core CCA1–LHY/TOC1 [30–33]. The interaction between *CPD* and *CCA1* may hold clues to the causes of the late-flowering phenotype of *cpd* mutants [12,34]. Furthermore, it has been reported that BR can modulate circadian rhythms and promote the periodicity of the circadian clock genes *CHLOROPHYLL A/B BINDING PROTEIN* (*CAB2*), *COLD AND CIRCADIAN-REGULATED 2* (*CCR2*) and *CCA1* [35]. This interaction is consistent with the observation that the period of *CCR2* is prolonged in *cpd* mutants [35]. Thus, the above findings suggest that BR regulates flowering time through the circadian clock system, a crucial mechanism in photoperiod pathway.

Soybean is a short-day crop of agricultural and economic importance. Soybean flowering is largely regulated by photoperiod, with many varieties highly photoperiod-sensitive. Typically, *Zigongdongdou* will not initiate flowering until short-day induction; this variety even undergoes flowering reversion in which the floral meristem developing the floral organs reverts to produce leaves when the photoperiod is altered from a short day to a long day [36–40]. This high sensitivity restricts the adaptability of soybean to diverse environmental conditions, limiting the season and region available to many high yield varieties, negatively impacting soybean production [41]. In addition, the photoperiod sensitivity is diverse among soybean varieties, leading to multiple maturity periods. Consequently, many varieties with good behavior cannot be hybridized with each other as a result of asynchronous florescence. Therefore, it has been long recognized by breeders that controlling flowering time is crucial to ensuring soybean yield [42].

In the current study, four soybean *CPD* homologous genes belonging to the BR biosynthesis pathway are found to be associated with soybean flowering. These GmCPDs are extremely similar with AtCPD in sequence and structure and can complement the AtCPD function in Arabidopsis mutants deficient in AtCPD. The expression levels of these *GmCPDs* all exhibit a sudden peak upon floral meristem initiation in soybean and are increased in a photoperiod-insensitive soybean variety, suggesting a relationship between BR biosynthesis genes and floral transition.

## Materials and Methods

### Plant Growth Conditions

Soybean varieties *Williams 82* and *Zigongdongdou* were grown in a chamber at day/night temperatures of 26/24°C. *Zigongdongdou* and *Heihe27* used for the analysis of *GmCPDs* expression in soybean varieties with different photoperiod sensitivities were grown at the temperature of constant 25°C. *Williams 82* plants were cultivated under a short-day condition (12/12 h day/night cycle). *Zigongdongdou* and *Heihe27* plants were cultivated under either short-day or long-day (16/8 h day/night cycle) conditions depending on the experiment.

Arabidopsis accessions Col-0 and *cpd-91* were grown at 22°C under a long-day condition (16/8 h day/night cycle) in potting soil or in half strength MS agar plates with 1% (w/v) sucrose. All plates were axenically cultured and packed with silver papers in the dark treatment.

### Brassinosteroid Treatment

In the BR response assay, 5-day-old *Williams 82* seedlings were cultivated in Hoagland solution after germination in soil. BR treatment was undertaken 10 days later by adding 1 μM 2,4-epibrassinolide (C28H48O6; TCR, Toronto, ON, Canada) to the solution. The treatment lasted for 2 hours, and the samples were collected every half hour.

In the root inhibition assay, Arabidopsis seeds were planted on vertically oriented plates containing half-strength MS medium supplemented with 1% sucrose in the absence or presence of 100 nM 2,4-epibrassinolide (24-epiBL). Root lengths were measured after seedlings were grown for 10 days.

### Sampling and RNA Isolation

The entire *Williams 82* plant was sampled for *GmCPDs* gene cloning. For tissue-specific expression analysis, hypocotyls, cotyledons and roots of *Williams 82* were collected from 7-day-old seedlings, and the leaves, stems and shoot apices were collected from 20-day-old adult plants. The flowers were tagged on the day of anthesis, and the pods were harvested when 0.5–2 cm long. After BR treatment, the leaves of *Williams 82* were collected every half hour and labeled 0.5 h (0.5 hour after treatment), 1 h, 1.5 h and 2 h. When the cotyledons of *Zigongdongdou* and *Heihe27* opened, SD (short-day), LD (long-day) photoperiod treatments were carried out. The SD13d-LD (transfer to an LD condition after a 13-day SD treatment) condition was applied only to *Zigongdongdou*. Plant leaves were collected every other day until the 25^th^ day following photoperiod treatment. Cotyledons were obtained at 3 d (3 days after photoperiod treatment), 6 d, and 9 d with leaves removed after cotyledon opening.

All samples were a mixture of more than five individual plants and were ground into powder in liquid nitrogen. Total RNA was extracted using TRIzol Reagent (Invitrogen, Carlsbad, CA, USA). The RNA from a whole Arabidopsis plant sample was isolated using the same method.

### Analysis of mRNA Expression Level by Real-Time PCR

cDNA for PCR was prepared using 1 μg of total RNA with a mixture of random primers. RT-qPCR analysis was performed on an ABI7900 instrument (Applied Biosystems, Foster City, CA, USA) using Takara SYBR Premix ExTaq (Takara, Shiga, Japan) for 40 cycles (95°C for 5 s; 60°C for 30 s; 72°C for 30 s). All reactions were carried out at least three times. Quantification of mRNA level was based on Ct (threshold cycle) values using a comparative Ct (2−ΔΔCt) method [43]. Data are presented as the mean±SD. The specific primers for each gene are shown in S1 Table.

### Vector Construction and Arabidopsis Transformation

The coding regions of *GmCPD1*, *GmCPD2*, *GmCPD3* and *GmCPD4* with additional *Xba*I and *Sac*I restriction sites were PCR-amplified. The *Xba*I–*Sac*I flanked *GmCPDs* fragments were cloned into the *Xba*I–*Sac*I sites of pTF101.1-GFP vector, replacing GFP and generating pTF101.1-GmCPD1, pTF101.1-GmCPD2, pTF101.1-GmCPD3 and pTF101.1-GmCPD4. These resulting constructs were verified by sequencing and restriction analysis and transformed into *Agrobacterium tumefaciens* strain GV3101. The *Agrobacterium*-mediated flower infiltration transformation method [44] was used to introduce *GmCPDs* into *cpd-91* Arabidopsis mutant plants. T1 generation seeds were harvested and selected on antibiotic-containing MS plates with 10 mg/L glufosinate ammonium (Sigma, St. Louis, MO, USA). Positive plants were confirmed by PCR analysis and propagated to obtain the T3 generation.

### Measurements and Statistical Analysis

All seedlings were axenically cultured on medium for light/dark analysis, BR treatment assays and leaf morphology analysis were scanned using an Epson perfection V700 photo scanner (Epson, Nagano, Japan). The images were analyzed using WinRHIZO Pro v.2009c software (Regent Instruments, Montreal, QC, Canada). For light/dark analysis, the hypocotyl lengths of 6-day-old seedlings were measured. Similarly, when the seedlings grown on medium with or without 24-epiBL in the BL treatment assay, the total root lengths of 10-day-old seedlings, hypocotyl length of 6-day-old seedlings and petiole length of 13-day-old seedlings were measured; the number of lateral roots of 10-day-old seedlings was also counted. For leaf morphology analysis, the first true leaves of 13-day-old seedlings were cut off at the bottom of the petioles and flattened on agar plates for scanning. Traits including petiole length, leaf area, length and width of the leaf blade were examined. Silique length and plant height were measured using a millimeter-graduated ruler. All measurements were repeated three times independently, and 30–50 seedlings were measured each time. Data are presented as the mean±SD and were subjected to Student’s t test with a sample size of 30 to determine differences among the groups.

## Results

### Cloning and Sequence Analysis of *GmCPD* genes in *Glycine max*

Four soybean *CPD* homologs (*GmCPDs*) were obtained from the soybean translated NCBI nucleotide database by a BLAST search using the amino acid sequence of Arabidopsis CPD (GenBank accession No. XP_002873219) as a query. These predicted genes were then assigned names based on their correspondence with AtCPD. The four potential homologous proteins, GmCPD1 (GenBank accession No. XP_003545232.1), GmCPD2 (GenBank accession No. XP_003519393.1), GmCPD3 (GenBank accession No. XP_003552845.1) and GmCPD4 (GenBank accession No. XP_003538460.1), are predicted to be between 473 and 480 amino acids in length and all belong to the cytochrome P450 (CYP) family.

**Fig 1:**
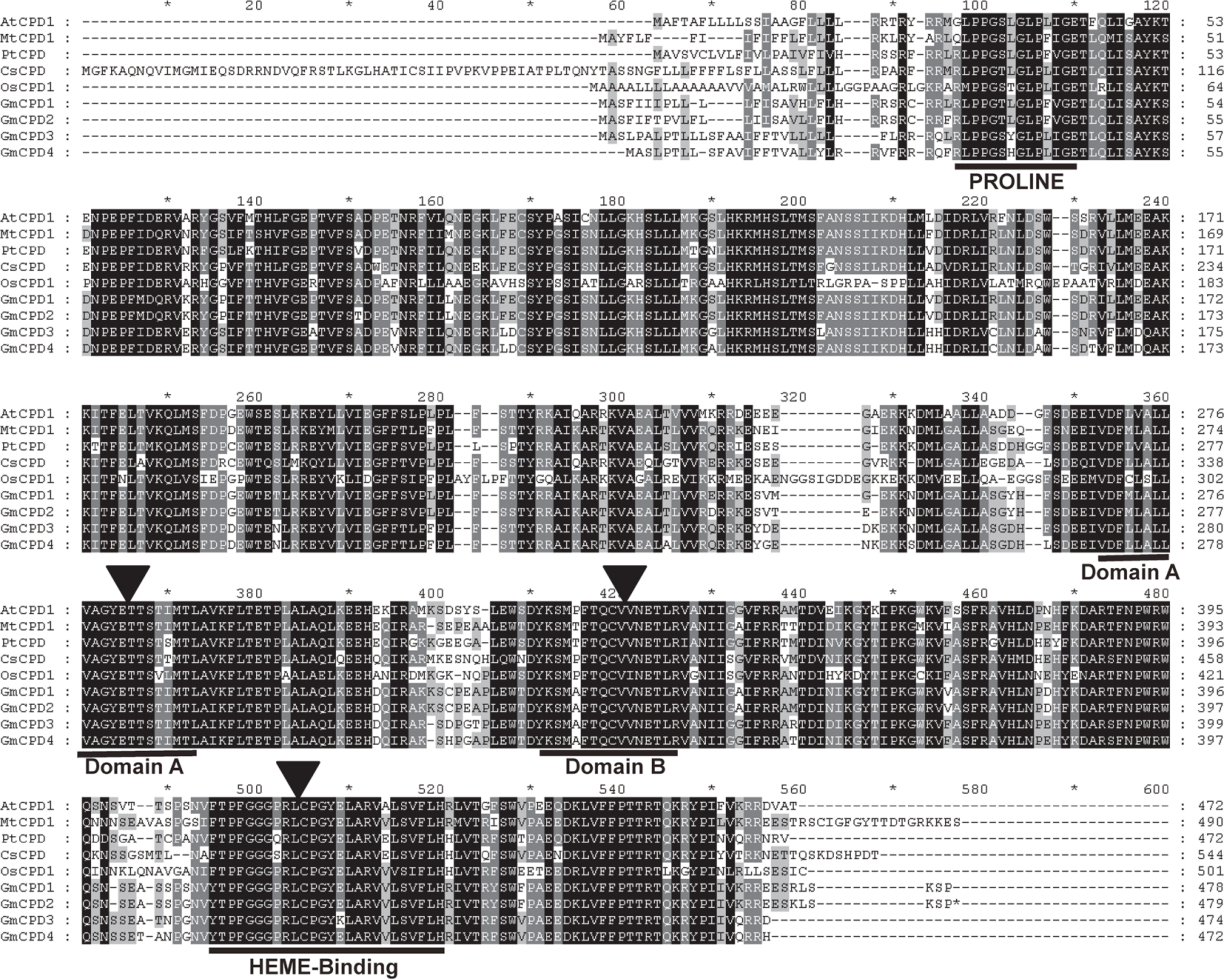
GmCPD amino acid sequence homology with CPDs from other species. Multiple alignments were performed by the Clustal W2 algorithm. Exact matches are boxed in black. Four domains found in cytochrome P450s: Proline-rich, Domain A (dioxygen-binding), Domain B (steroid-binding) and heme-binding are underlined. Arrowheads: Amino acid residues with important function. Accession numbers are as follows: AtCPD (XP_002873219), GmCPD1 (XP_003545232.1), GmCPD2 (XP_003519393.1), GmCPD3 (XP_003552845.1), GmCPD4 (XP_003538460.1), MtCPD1 (XP_003616626), PtCPD (XP_002311214), CsCPD (XP_004149251), OsCPD1 (NP_001066117).

The deduced amino acid sequences of the GmCPDs share 82–97% identity with each other and exhibit high similarity to the Arabidopsis CPD protein, with identities between 79% and 81% (Fig. 1). GmCPD1 has the highest identity of 81% while GmCPD2 has the lowest. An alignment of GmCPDs with known CPDs from other species reveals identities of 81–87% for MtCPD1 of *Medicago truncatula*, 76–80% for PtCPD of *Populus trichocarpa*, 75–77% for CsCPD of *Cucumis sativus*, and 59–63% for OsCPD1 of monocot *Oryza sativa* (Fig. 1). It is suggested that the amino acid sequences of CPDs are highly homologous across all species.

There are generally four structural domains in CPD proteins that exhibit catalytic features. The proline-rich region was thought to ensure the correct folding and proper orientation of the CPD protein. Domain A and domain B are involved in the dioxygen and steroid binding required for catalytic activity. The most characteristic P450 consensus sequence, the heme binding domain, is responsible for carbon monoxide binding ability [45,46]. As shown in Fig. 1, all CPDs contain these characteristic domains, and their amino acid sequences are highly conserved. There are only two amino acid differences between AtCPD and GmCPDs in the proline-rich region, one amino acid difference in domain B and at most two amino acid differences in the heme-binding domain. As for domain A, AtCPD and GmCPDs share 100% amino acid sequence identity (Fig. 1). Based on these findings, GmCPDs bear a striking similarity to AtCPD in sequence and structure, a trait that might imply functional similarity.

**Fig 2:**
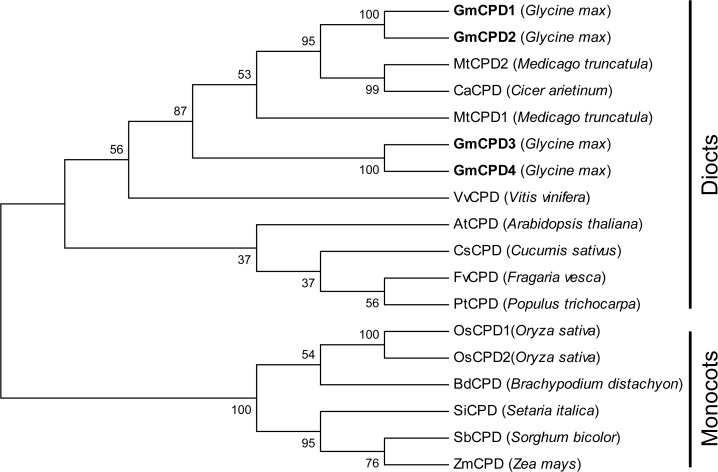
Phylogenetic tree of CPD proteins by neighbor-joining method using MEGA 5.02 software. Accession numbers are as follows: AtCPD (XP_002873219), GmCPD1 (XP_003545232.1), GmCPD2 (XP_003519393.1), GmCPD3 (XP_003552845.1), GmCPD4 (XP_003538460.1), MtCPD1(XP_003616626), MtCPD2(XP_003600878), CaCPD(XP_004490985), CsCPD (XP_004149251), FvCPD (XP_004307639), PtCPD (XP_002311214), VvCPD (XP_002270553), OsCPD1 (NP_001066117), OsCPD2 (NP_001065721), BdCPD (XP_003578946), SiCPD (XP_004978643), SbCPD (XP_002450249), ZmCPD (NP_001140596).

Phylogenetic analysis was performed using the deduced amino acid sequences of GmCPD and a range of putative CPDs from higher plants. The tree is clearly divided into two major clades: one clade corresponds to monocots, while the other clade corresponds to dicots (Fig. 2). The four GmCPDs all fall in the latter clade (Fig. 2). GmCPD1 and GmCPD2 are clustered together with *Medicago truncatula* and *Cicer arietinum*, while GmCPD3 and GmCPD4 branch off from the legume sub-clade (Fig. 2). The four GmCPDs all cluster relatively closely with AtCPD (Fig. 2), indicating that these proteins may have inherited more ancestral characteristics.

**Fig 3:**
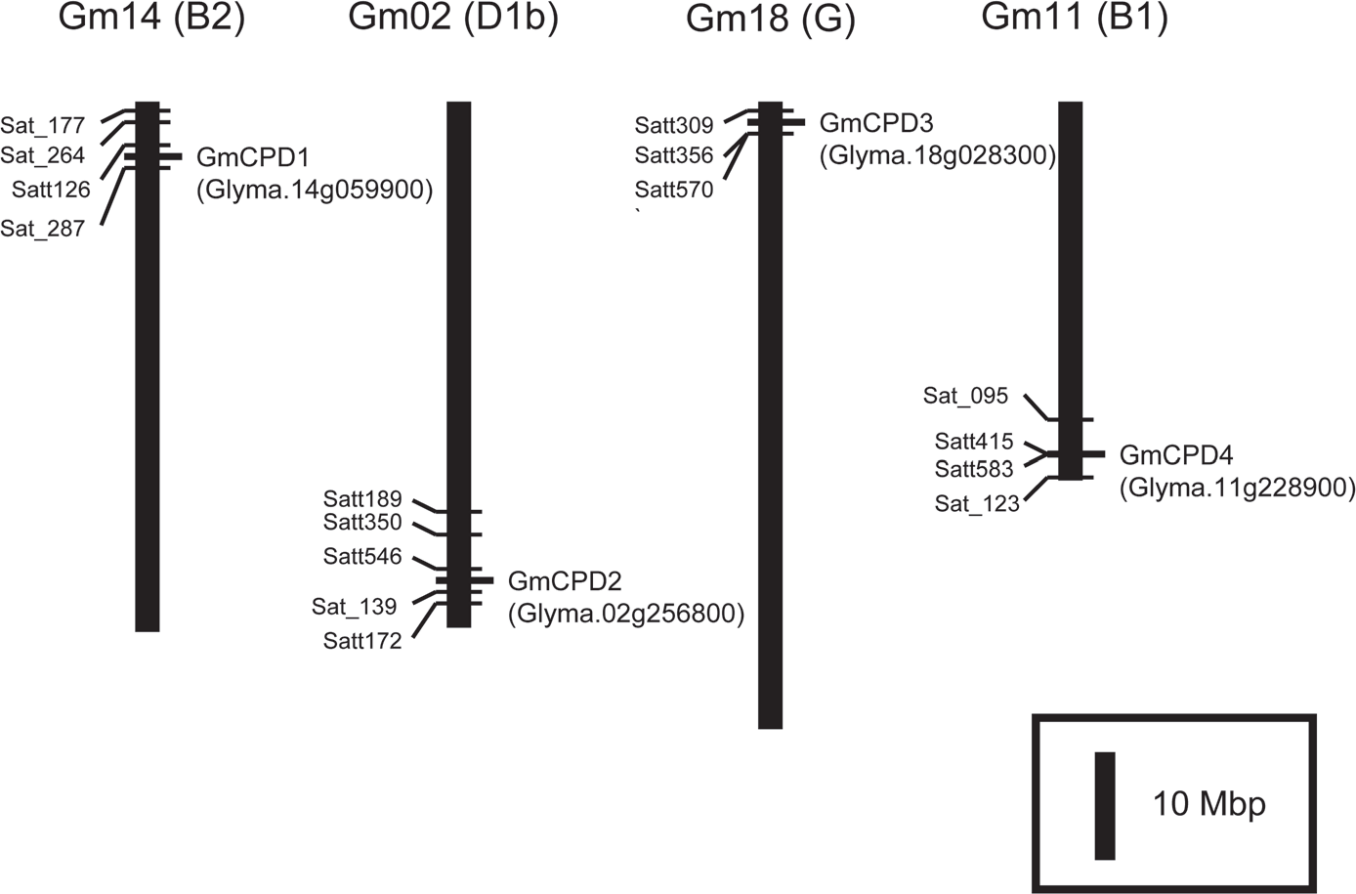
The genomic location of four *GmCPDs* on soybean physical map. Distance along the vertical bars indicates the physical distance reported in the Soybase and Phytozome database. The SSR markers near to *GmCPDs* location were also labeled on the corresponding positions of chromosome.

Genomic location of each *GmCPD* was targeted on physical map of soybean (*Glycine max*) genome based on the information on SoyBase (http://www.soybase.org) and Phytozome database (http://phytozome.jgi.doe.gov). They are all located in separate chromosome: *GmCPD1* (Glyma.14g059900), *GmCPD2* (Glyma.02g256800), *GmCPD3* (Glyma.18g028300) and *GmCPD4* (Glyma.11g228900) are located in Gm14 (B2), Gm02 (D1b), Gm18 (G) and Gm11 (B1), respectively (Fig. 3). There were not many SSR markers around *GmCPDs*. Around *GmCPD1*, Sat_177 and Sat_264 are associated with the QTLs of flower number; Satt126 is associated with lodging and Sat_287 also relates to seed coat color (Fig. 3). As for *GmCPD2*, Satt189, Satt350 and Satt546 are associated with the QTLs of first flower; Satt189 and Satt350 are associated with leaflet shape and leaf area respectively; Satt546 is associated with internode length; Sat_139, Satt546 and Satt172 are associated with the seed quality trait (Fig. 3). Satt309, Satt356 and Satt570 locate closely to *GmCPD3*: Satt309 and Satt356 associated with the QTLs of pod maturity; Satt356 is linked with internode length; Satt570 is associated with seed protein, lateral root density and root width (Fig. 3). Around the location of *GmCPD4*, Satt415 is associated with the internode length; Satt583 is associated with the length of reproductive stage; Sat_123 is associated with pod maturity and lodging; Sat_123, Satt583 and Sat_095 are all associated with seed weight (Fig. 3). Above all, the four *GmCPD* homologous are associated with the QTLs related to main aspects of soybean development.

### Expression Patterns of *GmCPDs* in Soybean

**Fig 4:**
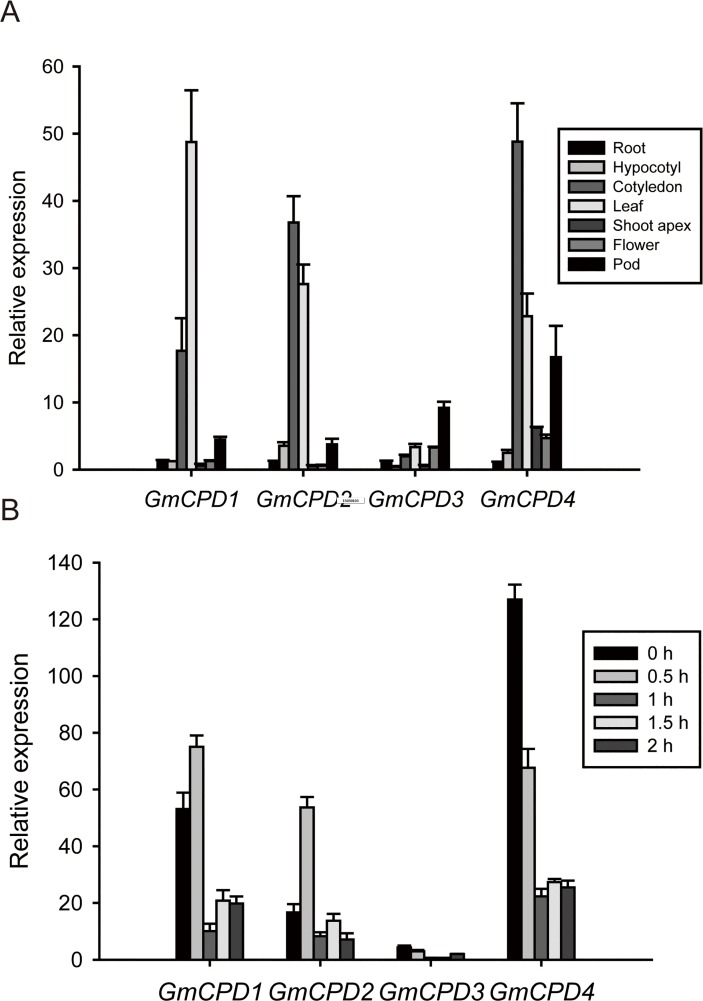
*GmCPDs* expression patterns in soybean. (**A**) Tissue-specific expression patterns of *GmCPDs* in soybean. The sampling time of each tissue is described in section 4 of the Materials and Methods (**B**) Inducible expression of four *GmCPD* genes in the soybean leaves under BR treatment. The relative expression levels are normalized to *GmG6PDH* (GenBank accession No. XM_003547631). The data represent the mean ± SD of three independent experiments.

Tissue-specific expression patterns of *GmCPDs* in soybean were systematically determined using RT-qPCR. These four *GmCPDs* are widely expressed in plant tissues but display different patterns. Although *GmCPD1*, *GmCPD2* and *GmCPD4* all showed higher expression levels in cotyledons and leaves, *GmCPD2* and *GmCPD4* had the highest level in cotyledons while *GmCPD1* had the highest level in leaves (Fig. 4A). These results are consistent with the expression pattern of *CPD* in Arabidopsis [47]. However, *GmCPD3* is an exception, exhibiting the highest mRNA accumulation in young pods but very low concentrations in other tissues (Fig. 4A). In addition, *GmCPD4* as well as *GmCPD1* and *GmCPD2* showed relatively high levels in young pods (Fig. 4A). These results are consistent with the important roles proposed for BRs in processes such as fruit development and ripening [48,49].

The BR sensitivity of *GmCPDs* was also tested in soybean. *William 82* adult plants were treated with 24-epiBL, and the leaf samples were collected every half hour. As shown in the RT-qPCR results, the expression levels of the four *GmCPDs* fluctuated, but overall the expression levels tended to decrease. The expression patterns of *GmCPD1* and *GmCPD2* were nearly equivalent but distinct from *GmCPD3* and *GmCPD4* (Fig. 4B). Following BR treatment, a sudden increase of *GmCPD1* and *GmCPD2* expression reached a maximum 0.5 h after treatment was initiated. *GmCPD1* and *GmCPD2* expression then sharply decreased, reaching a minimum approximately 1 h after treatment was started and subsequently increasing slightly to a plateau (Fig. 4B). In contrast, the expression levels of *GmCPD3* and *GmCPD4* rapidly decreased following BR treatment, reaching a minimum at 1 h and then leveling off (Fig. 4B). The above results indicate a highly sensitive response of *GmCPDs* to exogenous BR. Taken together with previous studies that show that *CPD* is feedback-inhibited by BR [13,47,50], our results corroborate the importance of *GmCPDs* in BR biosynthesis.

### Complementation of an Arabidopsis CPD-Deficient Mutant phenotype by *GmCPDs* Expression

**Fig 5:**
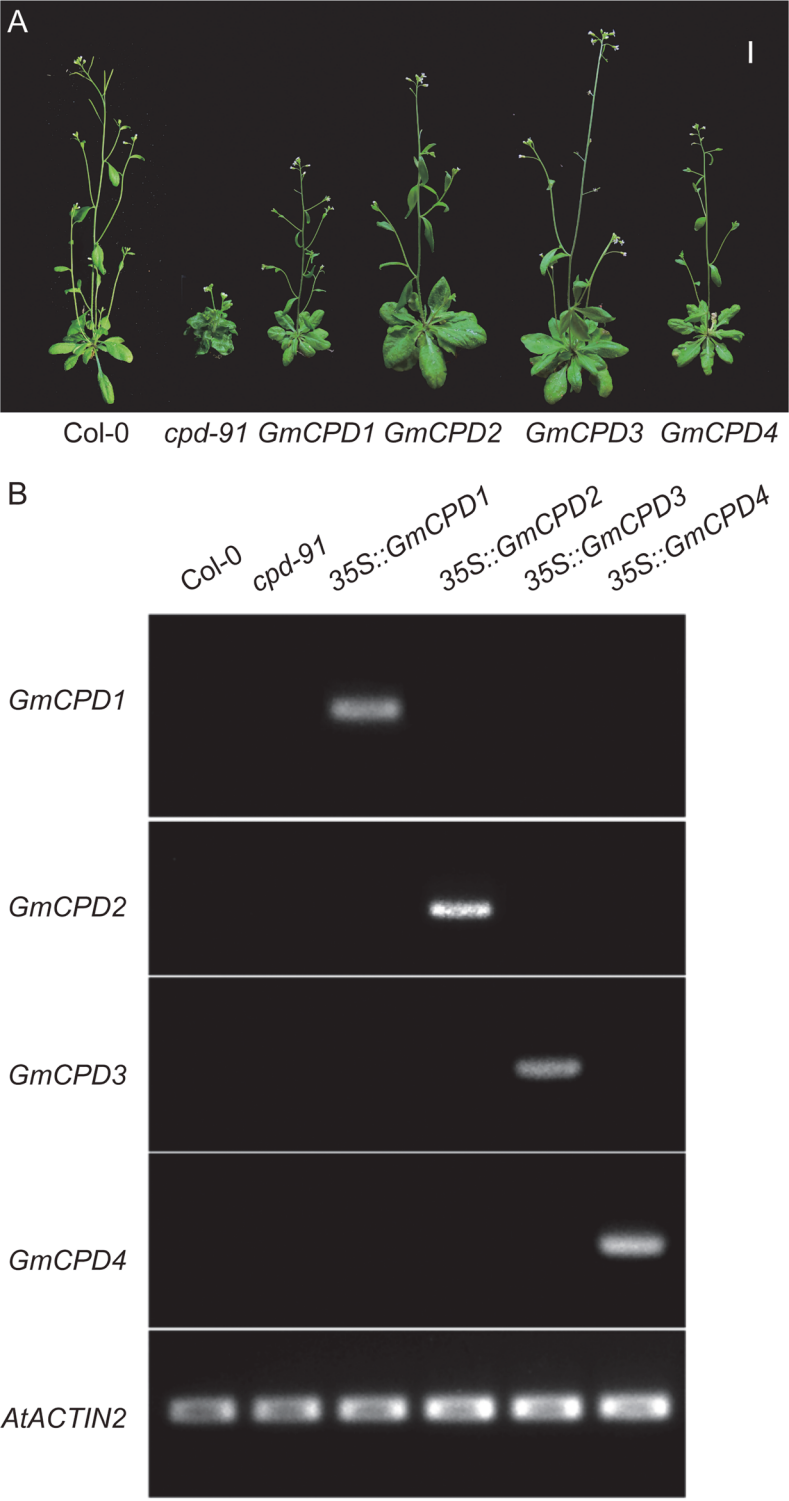
Four *GmCPDs* complement the phenotype of an Arabidopsis CPD-deficient mutant. (**A**) Phenotype comparison of adult plants. Bar = 10 mm; Col-0: wild type plants; *cpd-91*: CPD-deficient mutant; *GmCPD1*: 35S::*GmCPD1*; *GmCPD2*: 35S::*GmCPD2*; *GmCPD3*: 35S::*GmCPD3*; *GmCPD4*: 35S::*GmCPD4*; (**B**) RT-PCR analysis to detect *GmCPD* genes using specific primers described in S1 Table with *AtACTIN2* (GenBank accession No. AT3G18780) as a reference gene.

To test whether the *GmCPDs* can function in BR biosynthesis, the coding sequences of *GmCPD1*, *GmCPD2*, *GmCPD3* and *GmCPD*4 were placed under the control of a 35S constitutive promoter and introduced into a *cpd-91* mutant [13] of Arabidopsis. The goal was to evaluate whether the transgenes complement the mutant phenotype. Adult plants of *cpd-91*, a CYP90A1/CPD-deficient mutant, are small and dwarfed with rounded curled leaves. In contrast, the transgenic *GmCPD1*, *GmCPD2*, *GmCPD3* and *GmCPD*4 *cpd-91* mutant lines were all similar to the wild type in size, showing a rescue of the *cpd-91* mutant adult phenotypes (Fig. 5A). The RT-PCR results revealed that *GmCPD* genes can only be detected in the corresponding transgenic plants, indicating complementation by *GmCPDs* overexpression (Fig. 5B).

**Fig 6:**
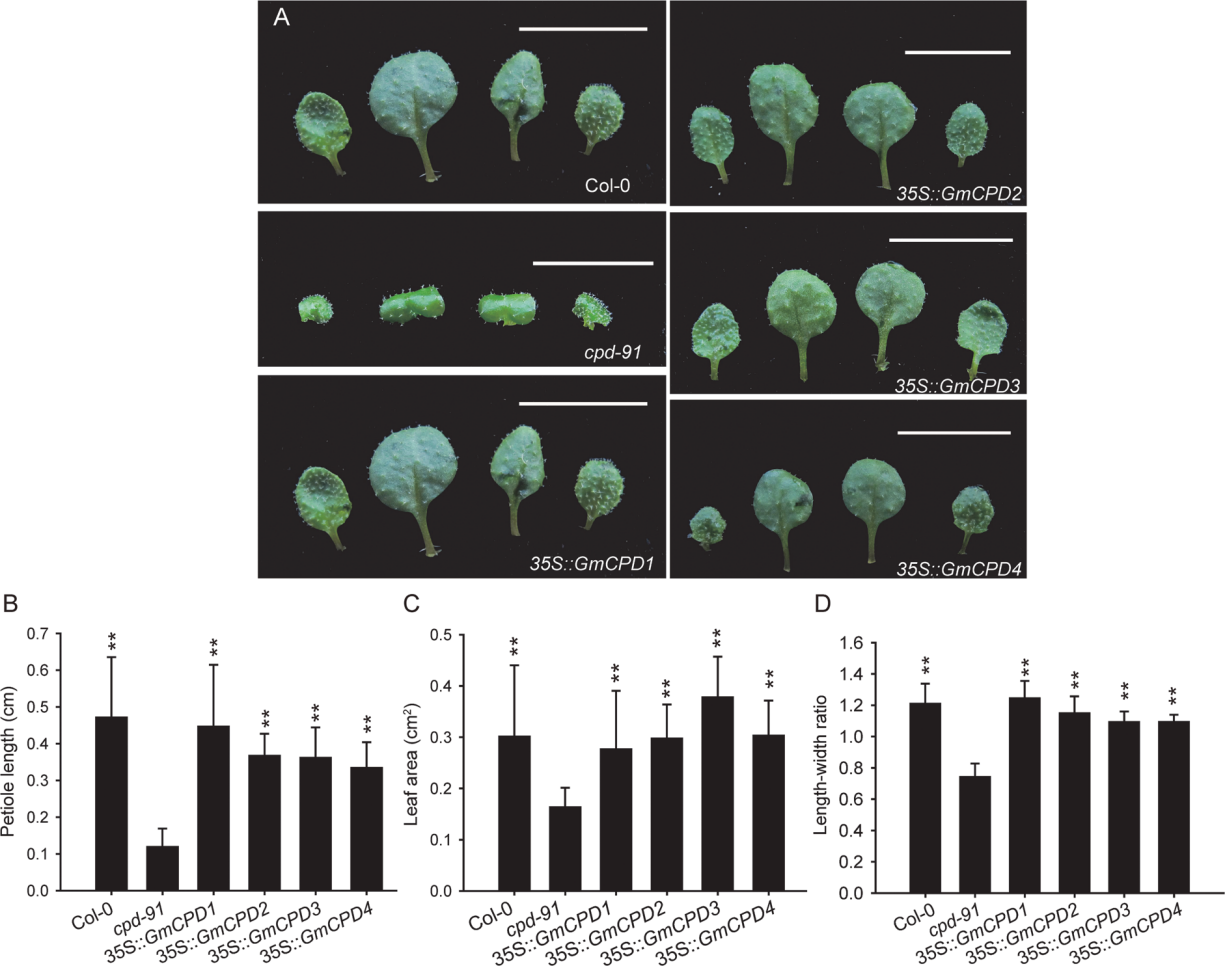
Comparison of leaf phenotype between the wild type (Col-0), CPD-deficient mutant (*cpd-91*) and transgenic plants. (**A**) Detailed leaf morphologies of 13-day-old Arabidopsis plants, Bar = 10 mm; (**B-D**) Statistical analysis of leaf measurements: the petiole length (**B**), leaf area (**C**) and length-width ratio (**D**). The data represents the mean ± SD of three independent experiments. The asterisks indicate significant differences compared to the *cpd-91* mutant (**, *P* < 0.01 by the *t*-test).

The leaf phenotypes of the transgenic lines all bear little resemblance to the *cpd-91* mutant, instead resembling the wild type phenotype (Fig. 6A). In a quantitative comparison, the mutant retained minimum values of petiole length (Fig. 6B), leaf area (Fig. 6C) and length-width ratio (Fig. 6D). The transgenic *GmCPD1*, *GmCPD2*, *GmCPD3* and *GmCPD*4 *cpd-91* lines were all similar to the wild type and were significantly different (*P* < 0.01) from the mutant (Fig. 6B-D).

**Fig 7:**
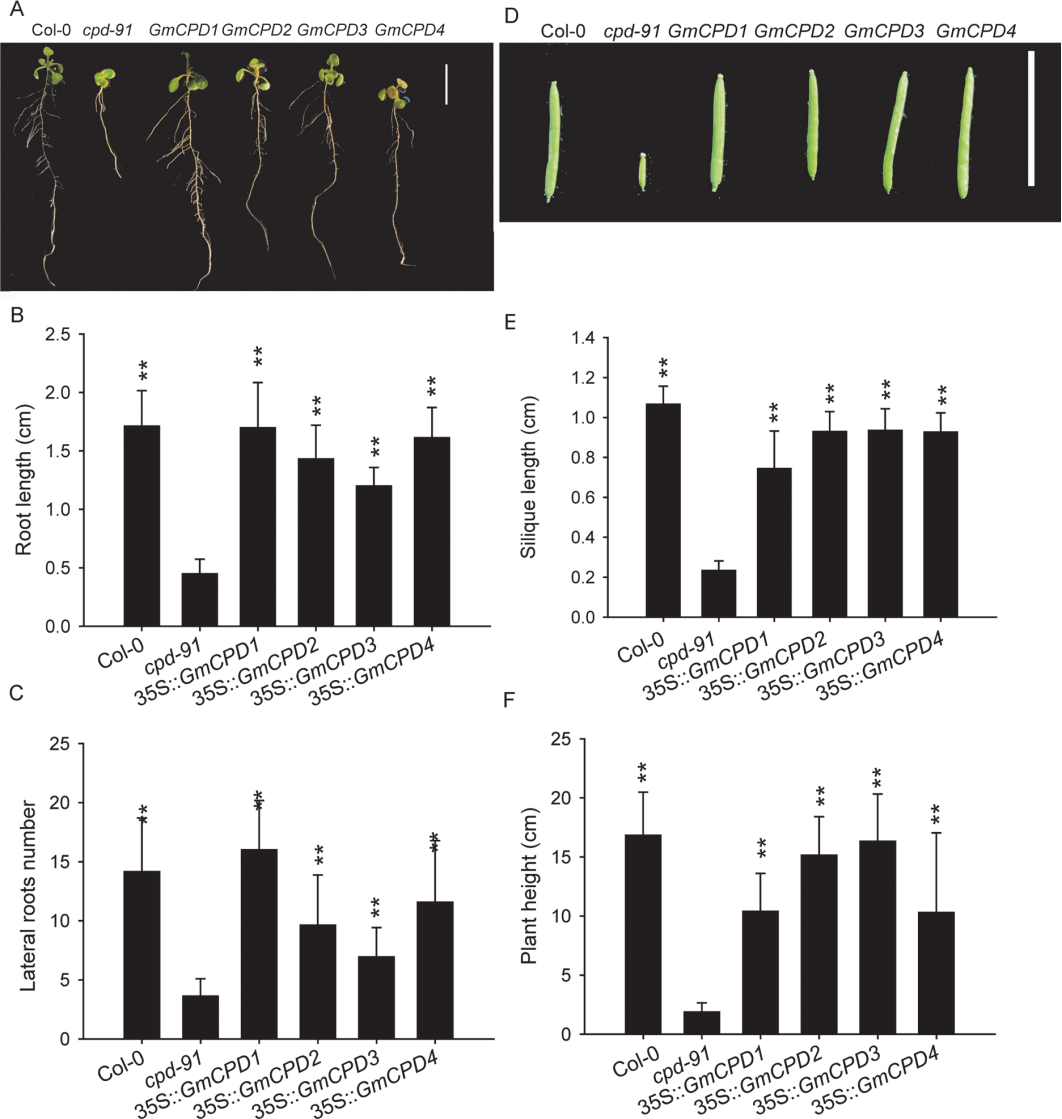
Phenotype comparison of root, silique and plant height between the wild type (Col-0), CPD-deficient mutant (*cpd-91*) and transgenic plants. (**A**) Root phenotype of 13-day-old Arabidopsis plants. Col-0: wild type plants, *cpd-91*: CPD-deficient mutant, *GmCPD1*: 35S::*GmCPD1*, *GmCPD2*: 35S::*GmCPD2*, *GmCPD3*: 35S::*GmCPD3*, *GmCPD4*: 35S::*GmCPD4*, Bar = 10 mm; (**B and C**) Total root length (**B**) and the lateral root number (**C**) of plants shown in A; (**D**) Silique morphology comparison. Bar = 10 mm; (**E and F**) Measurement and statistical analysis of the silique length (**E**) and plant height (**F**). The data represents the mean ± SD of three independent experiments. The asterisks indicate significant differences compared to the *cpd-91* mutant (**, *P* < 0.01 by the *t*-test).

Without the CYP90A/CPD gene, the morphological change in the roots was quite significant in the *cpd-91* mutant, with a small root length and undeveloped lateral roots (Fig. 7A-C). Conversely, all transgenic lines exhibited developed root systems that were similar to the wild type plants (Fig. 7A). Student’s *t*-tests indicated a significant difference (*P* < 0.01) between the transgenic lines and the mutant plants in root length and lateral root number (Fig. 7B-C).

The most obvious complementation of the mutant is the rescue of dwarfness. Similar to other BR mutants, *cpd-91* mutant is severely dwarfed. In contrast, the plant height of every transgenic line was remarkably higher (*P* < 0.01) than that of the mutant and very similar to that of the wild type (Fig. 7F).

The silique size of mutants is very small, only an average of 0.2 cm long and about twenty percent the length of Col-0 siliques (Fig. 7D, E). Each transgenic line was extremely distinct (*P* < 0.01) from the non-transformed mutant in silique size and resembled the wild type (Fig. 7E).

In conclusion, all four *GmCPDs* are functional and essential in leaf, root and plant type development.

### *GmCPD* Homologs Restore BR Biosynthesis in Arabidopsis *cpd-91* mutants

To further confirm that the rescue of the *cpd-91* mutant phenotype is due to a restored BR biosynthesis pathway via *GmCPDs* transformation, we tested the BR responses of complemented Arabidopsis compared with untransformed *cpd-91* and wild type Col-0.

**Fig 8:**
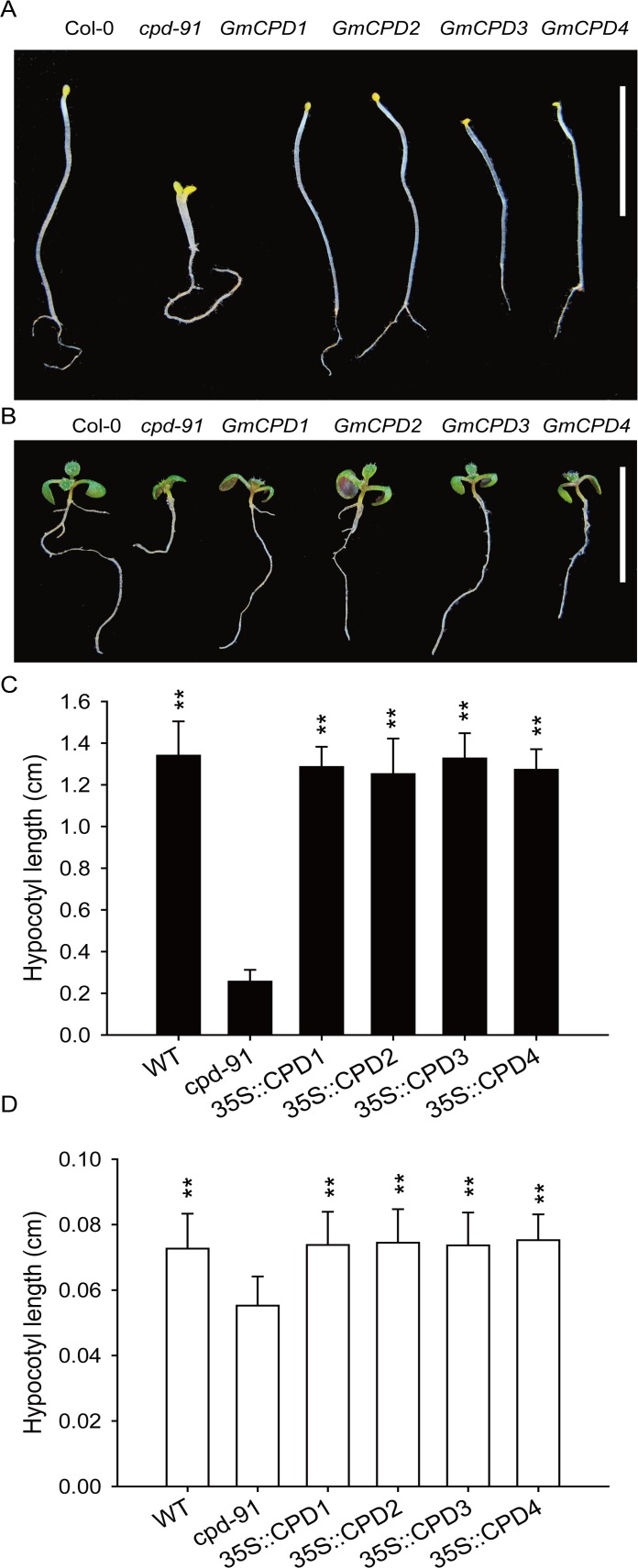
*GmCPDs* restore hypocotyl elongation of CPD-deficient Arabidopsis mutants in both light and darkness. (**A and B**) Morphologies of the five-day-old seedlings grown in darkness (**A**) and light (**B**). Col-0: wild type plants, *cpd-91*: CPD-deficient mutant, *GmCPD1*: 35S::*GmCPD1*, *GmCPD2*: 35S::*GmCPD2*, *GmCPD3*: 35S::*GmCPD3*, *GmCPD4*: 35S::*GmCPD4*, Bar = 10 mm; (**C and D**) Average hypocotyl length of seedlings in darkness (**C**) and light (**D**). The data represents the mean ± SD of three independent experiments. The asterisks indicate significant differences compared to the *cpd-91* mutant (**, *P* < 0.01 by the *t*-test).

6-day-old seedlings grown in light and darkness were screened for hypocotyl elongation during skotomorphogenesis and photomorphogenesis. In the dark, *cpd-91* mutant seedlings underwent constitutive photomorphogenesis, exhibiting short hypocotyls and open cotyledons (Fig. 8A, C). In contrast, the transgenic lines and wild type exhibited longer hypocotyls and closed apical hooks (Fig. 8A, C). When grown in the light, *cpd-91* mutant seedlings exhibited shorter hypocotyls than the wild type (Fig. 8B, D). This mutant phenotype was complemented by all four transgenes (Fig. 8B, D). Student’s *t*-tests indicate a significant difference (*P* < 0.01) in hypocotyl length between the transgenic lines and the mutant plants in both the light and darkness (Fig. 8C, D).

**Fig 9:**
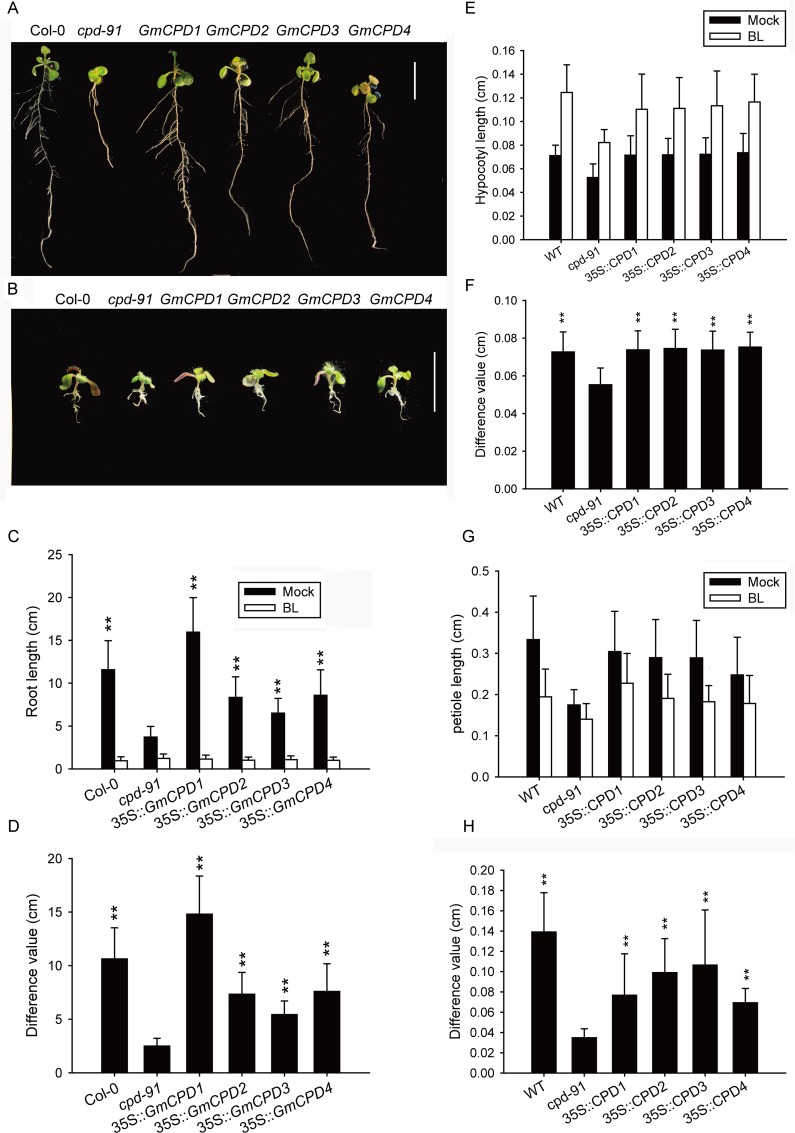
The variation in the length of root, hypocotyl and petiole showing different brassinosteroid (BR) responses in the wild type (Col-0), CPD-deficient mutant (*cpd-91*) and transgenic Arabidopsis plants. (**A and B**) Phenotypes of the wild type, CPD-deficient mutant and transgenic plants in half-strength MS medium supplemented with (**B**) or without 100 nM 24-epiBL (**A**). Col-0: wild type plants, *cpd-91*: CPD-deficient mutant, *GmCPD1*: 35S::*GmCPD1*, *GmCPD2*: 35S::*GmCPD2*, *GmCPD3*: 35S::*GmCPD3*, *GmCPD4*: 35S::*GmCPD4*. Bar = 10 mm; (**C**) Measurements of the total root length of the seedlings in the root inhibition assay; (**D**) Root length shortening after BR treatment; (**E**) Hypocotyl length of 6-day-old seedling in medium with or without 24-epiBL; (**F**) The elongation of hypocotyl after BR treatment; (**G**) Petiole length of 13-day-old seedling with or without BR treatment; (**H**) Petiole length shortening after BR treatment. The data represents the mean ± SD of three independent experiments. The asterisks indicate significant differences compared to the *cpd-91* mutant (**, *P* < 0.01 by the *t*-test).

In the root growth inhibition assay, 10-day-old complemented Arabidopsis, *cpd-91* mutants and the wild type were grown on 1/2 MS medium containing 100 nM 2,4-epibrassinolide (24-epiBL). All seedlings showed shortened roots in response to 24-epiBL but behaved differently in root shortening (Fig. 9A-D). The transgenic lines and the wild type displayed greater shortening than *cpd-91* plants (Fig. 9D), indicating a stronger response to BR.

Additionally, under the treatment of 100 nM 24-epiBL, all the seedlings exhibited elongated hypocotyl and shortened petiole (Fig. 9E-H). Compared to transgenic lines and Col-0 Arabidopsis, *cpd-91* mutant showed the shortest length of hypocotyl and petiole both in BL treatment and normal conditions (Fig. 9E, G). The transgenic lines resembled the wild type and displayed greater hypocotyl elongation and petiole shortening than *cpd-91* mutant (Fig. 9F, H).

Therefore, physiological response phenotypes of mutant plants are complemented by *GmCPDs* expression, suggesting a restored BR biosynthesis pathway in transgenic lines. This result further demonstrates that the *CPD* homologous genes in soybean, *GmCPD1*, *GmCPD2*, *GmCPD3* and *GmCPD4*, are functional in the BR pathway.

### *GmCPDs* Are Involved in Floral Regulation of Arabidopsis

**Fig 10:**
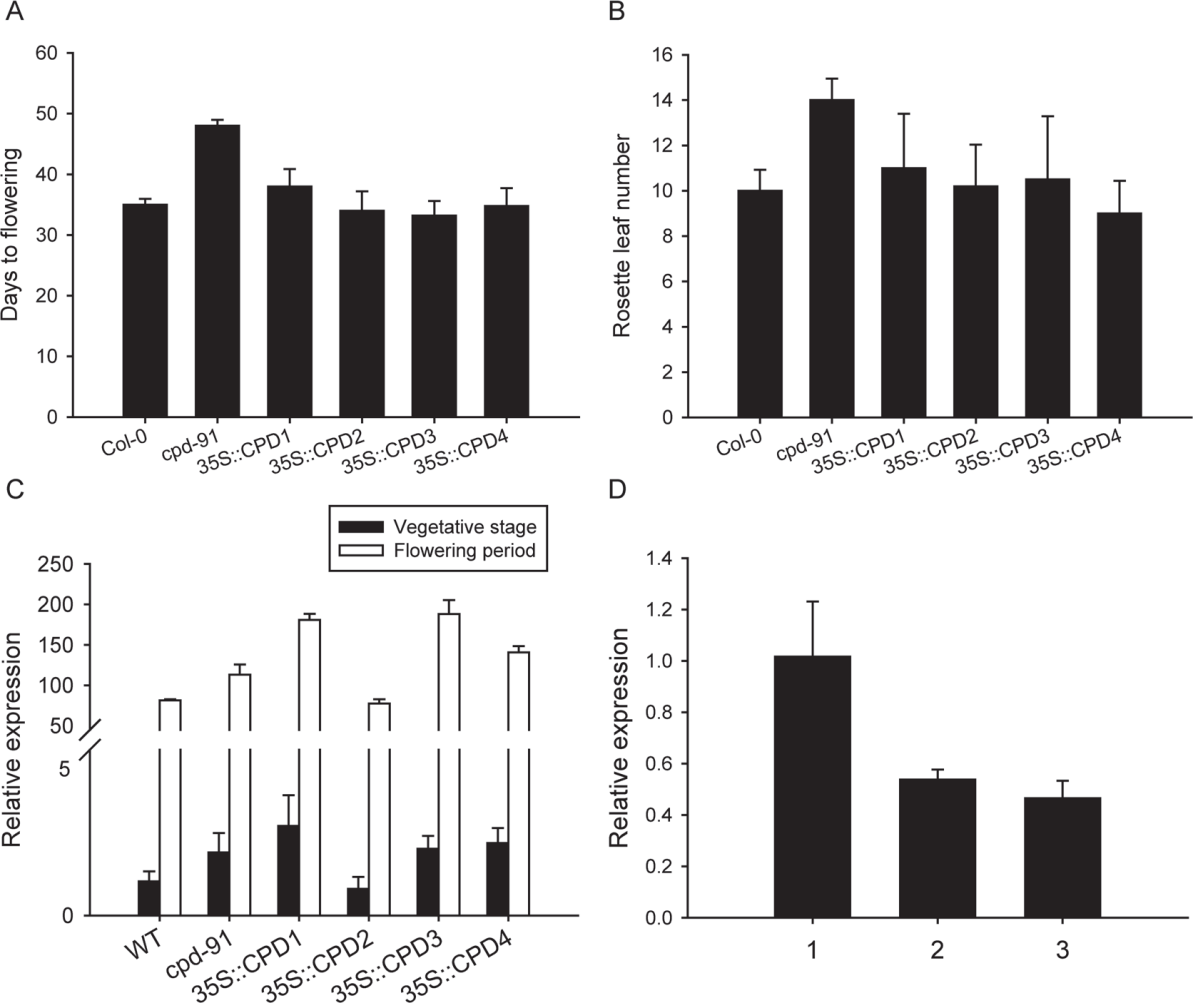
Complementation of late-flowering phenotype, expression pattern during flowering and effects on flowering related gene were indicated the roles of *CPD* in Arabidopsis flowering. (**A and B**) Flowering time analysis among wild type (Col-0), CPD-deficient mutant (*cpd-91*) and transgenic Arabidopsis plants. Comparison of the days to flowering (**A**) and the rosette leaf number (**B**) at anthesis; (**C**) The expression pattern of Arabidaopsis *FLOWERING LOCUS T* (*AtFT*) in vegetative stage and flowering period; (**D**) The expression pattern of *AtCPD* during flowering. 1, 2 and 3 represent three developmental stages. 1: vegetative stage, two weeks after emergence; 2: flowering initiation, the time that inflorescence-bud just emerged; 3: flowering period, one week after flowering.

The above results show the phenotypic rescue of Arabidopsis CPD-deficient mutant by *GmCPDs* expression. In addition to rescuing morphology, overexpression of *GmCPDs* also complemented the delayed flowering of the *cpd-91* mutant. The observation that the *cpd-91* mutant flowered approximately 10 days later than the Col-0 wild type is in agreement with previous observations (Fig. 10A, B). The transgenic plants transformed with any of the four *GmCPD* homologs all bloomed simultaneously with the wild type plant, much earlier than the *cpd-91* plants (Fig. 10A, B).

In order to investigate the roles of *CPD* in flowering regulation, the expression pattern of flowering integrating gene, *Flowering Locus T* (*FT*), was examined in the transgenic plants compared with non-transformed *cpd-91* and Col-0 wild-types (Fig. 10C). *AtFT* acts as floral integrator of all four flowering pathways [51]. The *AtFT* product, which can move in long distance through the phloem to initiate flowering at the shoot apex, is a main determinant of the timing of flowering [52]. In our results, all the groups exhibited similar expression pattern that *AtFT* gene maintained at a very low level in the vegetative stage and expressed highly when flowered (Fig. 10C). Except for that the *35S*::*GmCPD2* transgenic line showed similar level of *AtFT* expression to the wild type, the other three transgenic lines expressed diversely but all higher than *cpd-91* mutants (Fig. 10C).

In addition, the expression pattern of *AtCPD* gene during flowering was also examined in Arabidopsis leaves that were collected in three developmental stages: vegetative growth (two-week-old), flowering initiation and flowering period (one week after beginning flowering). It is showed that the *AtCPD* transcripts were more abundant in vegetative stage, but decreased during flowering (Fig. 10D).

### The Potential Roles of *GmCPDs* in Soybean Flowering Regulation

To further study the roles of *GmCPDs* in flowering, *GmCPDs* transcript levels were tested in soybean, a typical short-day plant that can undergo flowering reversion. In a previous study by our lab, 13 days of SD treatment before transfer to an LD condition are enough for flowering reversion to occur in soybean var. *Zigongdongdou*. Based on this observation, an effective flowering reversion system was established. In this system, three developmental states, flowering, continuous vegetative growth and flowering reversion, can be observed in *Zigongdongdou* plants under different photoperiods (SD, LD, 13SD-LD). Genes related to photoperiodism and flower development are preferentially studied in this system. Accordingly, leaf samples were collected in each photoperiod, and the relative expression levels of *GmCPDs* were analyzed to investigate the potential roles of *GmCPDs* in flowering.

**Fig 11:**
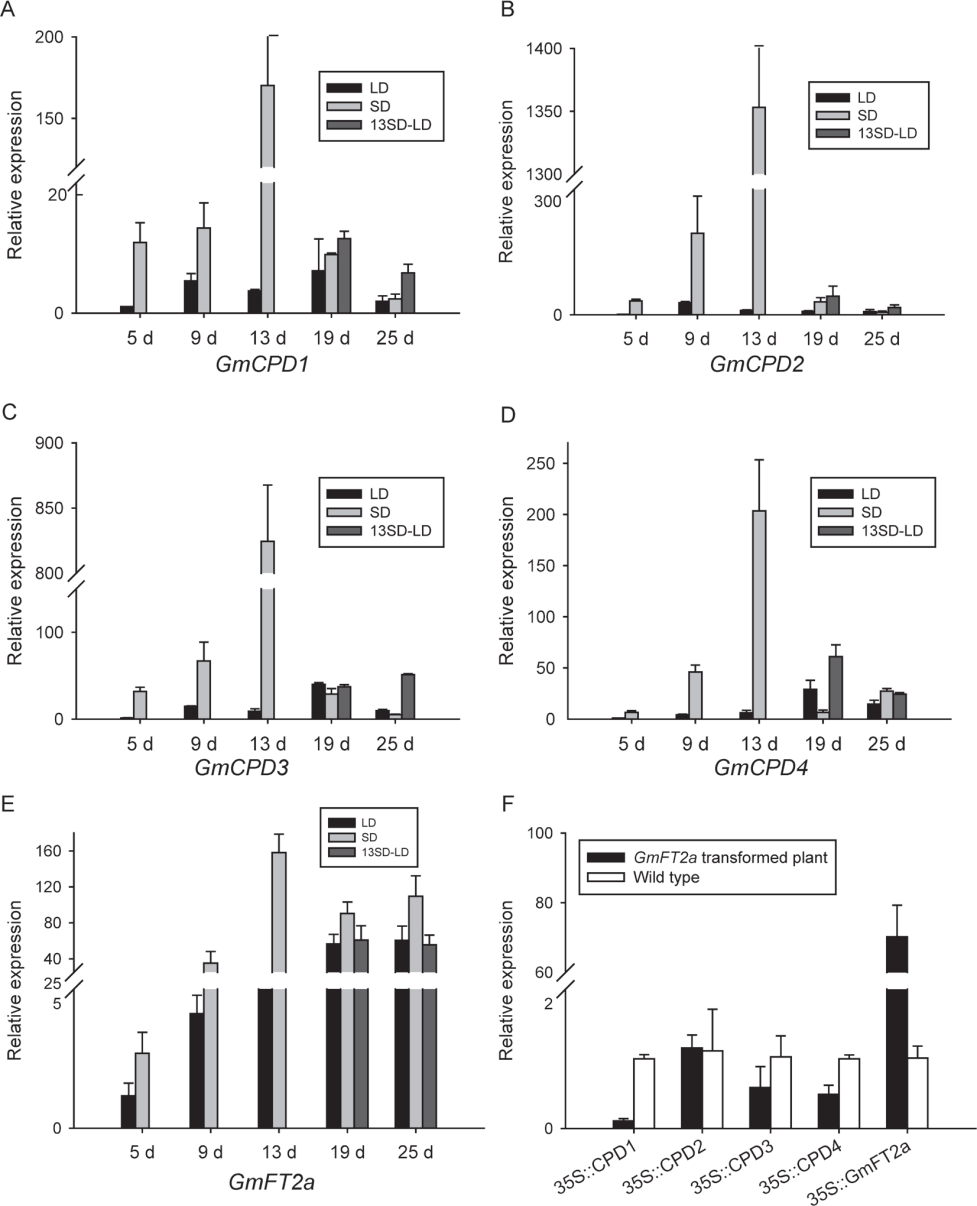
The expression pattern of *GmCPD* genes in the soybean flowering reversion system and *GmFT2a* transgenic soybean. LD: long day treatment; SD: short day treatment; 13SD-LD: transfer to long day conditions after 13 days of short day treatment; leaf samples of *Zigongdongdou* plants under each photoperiod were collected and qRT-PCR was used to analyze the expression of *GmCPD1* (**A**), *GmCPD2* (**B**), *GmCPD3* (**C**), *GmCPD4* (**D**) and *GmFT2a* (**E**); (**F**) The expression of *GmCPDs* in *GmFT2a* transgenic soybean plants compare to non-transformed *Zigongdongdou*. The *x*-axis represents the days of treatment. Relative expression levels are normalized to *GmG6PDH* (GenBank accession No. XM_003547631). The data represent the mean ± SD of three independent experiments.

As shown in the results, all *GmCPDs* have the same expression pattern: expression was maintained at a much lower level in the LD condition (Fig. 11A-D). Conversely, when treated in SD, *GmCPDs* expression levels were gradually elevated at first. Once SD treatment reached the 13^th^ day, *GmCPDs* levels sharply increased to a maximum, then decreased suddenly under both the SD and LD conditions (Fig. 11A-D). Obviously, these results suggest that *GmCPDs* expression is under photoperiod control and is upregulated by SD, a day length that induces flowering. Interestingly, the expression quantity of *GmCPDs* on the 13^th^ day is around tenfold that of the 9^th^ day and from nineteen to forty-five times that of the 19^th^ day (Fig. 11A-D). The peak on the 13^th^ day is so sharp that we cannot help but wonder what happens on this day. It was found in a previous study that the apical meristem of *Zigongdongdou* begins to initiate floral primordia on the 13^th^ day of SD treatment [39]. The above results suggest a certain relationship between *GmCPDs* and floral initiation through the photoperiod pathway.

When plants were grown in LD after SD induction, the expression of *GmCPDs* all decreased but still higher than that of plants grown in either continuous LD or SD (Fig. 11A-D). This result was not consistent with the expression pattern of *GmFT2a*, an integrator in photoperiod pathway. The expression of *GmFT2a* maintained in a rather low level in either LD or vegetative stage and raised around the 13^th^ day in SD when flowering initiated (Fig. 11E). When returned to the LD condition, *GmFT2a* expression decreased to the same level of that in the continuous LD treatment (Fig. 11E). Unlike *GmFT2a*, the expression of *GmCPDs* had additive effect that the SD effects could be accumulated when turned into LD condition, suggesting the distinct roles of *GmCPDs* in flowering regulation.

Since there was no obvious effect on the pattern of *AtFT* expression in the absence of CPD (Fig. 10C) and expression patterns between *GmFT2a* and *GmCPDs* in flowering reversion were different (Fig. 11A-E), it might imply that no direct interaction between *GmFT2a* and *GmCPDs*. To text this, the expression of *GmCPDs* expression was examined in *GmFT2a* transgenic soybean and compared with the non-transformed *Zigongdongdou* (Fig. 11F). The published data by our lab [53] have showed that one line of *GmFT2a* transgenic *Zigongdongdou* flowered approximately 20 days after emergence under non-inductive LD conditions. The expression level of *GmCPDs* in this line was found to be maintained in a quite low level and even decreased compared to the wild type on the occasion that *GmFT2a* expressed extremely high (Fig. 11F). Therefore, the involvement of *GmCPDs* in flowering regulation may not be linked to the direct interaction with *GmFT2a*.

### *GmCPDs* Expression in Soybean Varieties with Different Photoperiod Sensitivities

**Fig 12:**
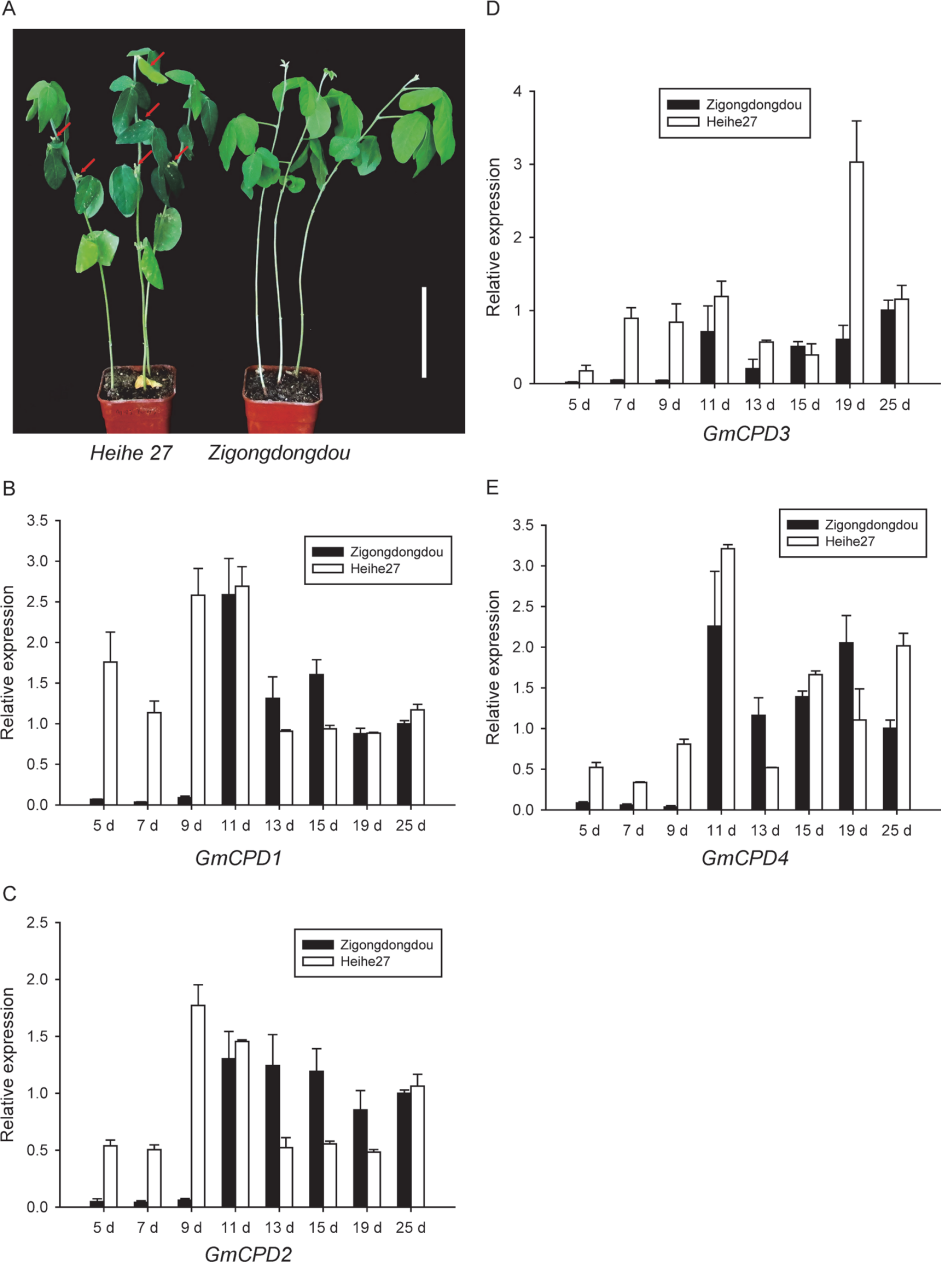
Expression patterns of *GmCPDs* in soybean varieties of different photoperiod sensitivities (*Zigongdongdou* and *Heihe27*) during LD treatment. (**A**) The phenotype of early and late flowering soybean plants. *Heihe 27* on the left is a photoperiod-insensitive early-maturity variety; *Zigongdongdou* on the right is a photoperiod-sensitive late-maturity variety. The photo was taken at the 36^th^ day after emergence under long day condition. *Heihe 27* had already produced one pod with length more than 2 cm and some younger pods (labeled by red arrows), while *Zigongdongdou* remained vegetative growth. Bar = 10 cm; Leaf samples from long day grown soybean plants were taken at the 5^th^, 7^th^, 9^th^, 11^th^, 13^th^, 15^th^, 19^th^ and 25^th^ day after cotyledon opening and labeled as 5 d, 7 d, 9 d, 11 d, 13 d, 15 d, 19 d and 25 d; qRT-PCR was carried out to determine the relative expression levels of *GmCPD1* (**B**), *GmCPD2* (**C**), *GmCPD3* (**D**) and *GmCPD4* (**E**) in each sample. Relative expression levels are normalized to *GmG6PDH* (GenBank accession No. XM_003547631); the data represent the mean ± SD of three independent experiments.

Soybean varieties are diverse in photoperiod sensitivity. *Zigongdongdou* is a photoperiod-sensitive late-flowering variety that only flowers under the SD condition. In contrast, the photoperiod-insensitive early-flowering variety *Heihe27* blooms approximately 25–27 days after emergence under both LD and SD conditions [54]. As it is shown in Fig. 12A, at the 36^th^ days after emergence, *Heihe 27* had already set pods while *Zigongdongdou* still underwent vegetative growth under the LD condition. These two typical varieties were chosen to evaluate the expression pattern of *GmCPDs* in soybean varieties with different photoperiod sensitivities.

We screened leaf samples from *Zigongdongdou* and *Heihe27* after various days of LD treatment. In the 5^th^, 7^th^ and 9^th^ day after LD treatment (5 d, 7 d and 9 d), all *GmCPD* genes were expressed at very low levels in *Zigongdongdou* but at extremely high levels in *Heihe27* (Fig. 12). After the 11^th^ day, the transcript levels of *GmCPDs* were remarkably upregulated in *Zigongdongdou* but slightly decreased and maintained in *Heihe27* (Fig. 12). The expression patterns of *GmCPD1*, *GmCPD2* and *GmCPD4* were nearly the same; the expression levels of these *GmCPDs* were obviously higher in *Heihe27* than *Zigongdongdou* from day 5 to day 11 d. From day 13 to day 19, the expression levels in *Zigongdongdou* were increased and higher than *Heihe27*, in which the levels were downregulated. At day 25, *Heihe27* had higher expression levels compared to *Zigongdongdou* (Fig. 12A, B and D). As for the *GmCPD3* gene, the expression levels in *Heihe27* were always higher than *Zigongdongdou* except for day 15. *GmCPD3* was most highly expressed in *Heihe27* at day 19 (Fig. 12C). However, the expressions of *GmCPDs* in *Zigongdongdou* under the LD condition in this experiment (Fig. 12) have differences with the results shown in Fig. 11A-D. This may due to the different culture temperature (described in section of Material and Methods) and sampling time. The leaf samples in this experiment were collected in the morning, while the samples in Fig. 11 were collected in the afternoon. Since genes usually have different expression levels during the day, the results in the two experiments are not comparable. We only analyzed the expression differences of *GmCPDs* among *Zigongdongdou* and *Heihe 27* in this experiment that carried out in the same condition and sampled at the same time every day.

**Fig 13:**
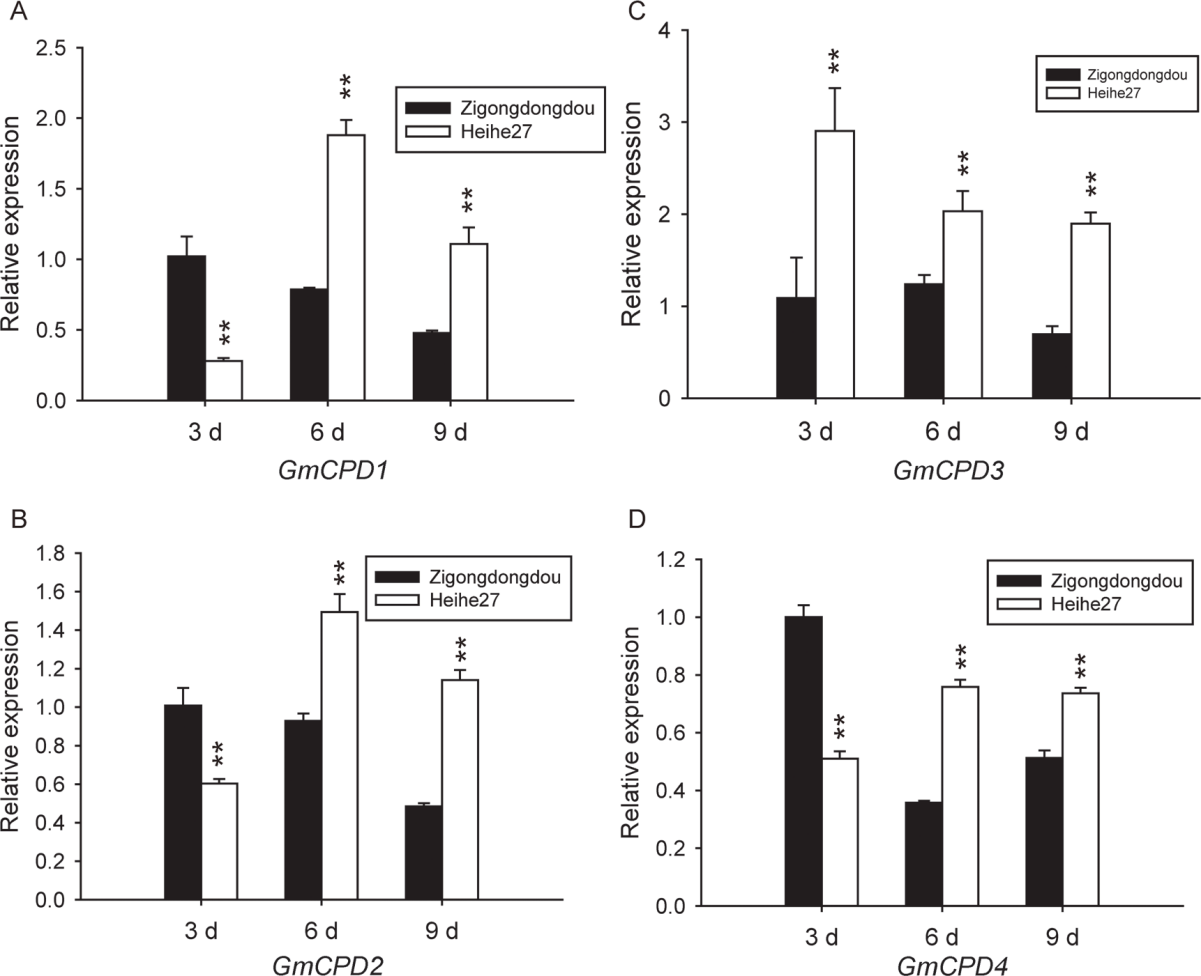
Comparison of *GmCPDs* expression in the cotyledons of *Zigongdongdou* and *Heihe27*, two soybean varieties with distinct photoperiod sensitivities. Relative expression levels of *GmCPD1* (**A**), *GmCPD2* (**B**), *GmCPD3* (**C**) and *GmCPD4* (**D**) were analyzed by qRT-PCR on the 3^rd^ day (3 d), 6^th^ day (6 d) and 9^th^ day (9 d) after cotyledon opening under the LD condition. Relative expression levels are normalized to *GmG6PDH* (GenBank accession No. XM_003547631). The data represent the mean ± SD of three independent experiments, and asterisks indicate significant differences compared to the *Zigongdongdou* plants (**, *P* < 0.01 by the *t*-test).

Leaves and cotyledons are the two main tissues in which *GmCPDs* are expressed (Fig. 4A). Therefore, cotyledons were also collected from *Zigongdongdou* and *Heihe27* on the 3^rd^, 6^th^ and 9^th^ days after LD treatment. *GmCPD1*, *GmCPD2* and *GmCPD4* had similar expression patterns: their expression levels tended to decreased with time in *Zigongdongdou* but increased in *Heihe27*. Although levels in *Zigongdongdou* were higher on the 3^rd^ day compared to *Heihe27*, the levels were much lower on the 6^th^ and 9^th^ days (Fig. 13A, B and D). The expression pattern of *GmCPD3* was rather special: the expression levels of *GmCPD3* in both *Zigongdongdou* and *Heihe27* decreased each day, but the gene was still expressed more highly in *Heihe27* compared to *Zigongdongdou* (Fig. 13C).

Regardless of whether leaves or cotyledons were measured, all *GmCPDs* exhibited more vigorous expression (much higher level) in *Heihe27* compared to *Zigongdongdou*, especially in the early days of LD treatment. The flowering of *Heihe27* is less regulated by photoperiod and can be initiated by the LD non-inducible day length. Compared to the strict short day flowering variety *Zigongdongdou*, the expression of *GmCPDs* is increased in *Heihe27*. In our opinion, differences in florescence between varieties is not only determined by the flowering regulation pathway but also by how each variety has been prepared for flowering; such preparation is affected by many factors, which may include *GmCPDs*.

## Discussion

### BR Intermediate Products Catalyzed by GmCPD Move Long Distances

In the tissue-specific expression assay, the observation that *GmCPDs* are expressed mainly in cotyledons and leaves (Fig. 4A) is consistent with the expression pattern of *CPD* in Arabidopsis [47] but does not coincide with the distribution pattern of bioactive BR [55,56]. The bioactive BR levels in vegetative tissues are much lower [30,56,57], with the highest levels generally occurring in reproductive organs [48,49,56,58], where BR can easily perform its intended function due to its lack of transport [30,55]. It has also been reported that the transcript levels of most BR biosynthesis genes are generally higher in tissues with high BR levels [48,49,57,59]; *CPD* obviously is an exception. Because CYP90A/CPD encoded by the *CPD* gene catalyzes an early step of BR synthesis [19], long-distance movements are required for BR intermediate products to finish synthesis where untransported bioactive BR are accumulated. With this assumption, the paradox that *GmCPDs* transcript levels are not higher in tissues with high BR levels is readily explained.

However, long-distance transformation is so costly that we wonder if the higher expression of *GmCPDs* in vegetative tissues holds further meanings. One possibility is because *CPD* is under light-dependent diurnal regulation primarily mediated by phytochrome signaling [23], leaves and cotyledons, where phytochrome collects, are preferred. In addition, recent work has revealed that BR plays a controlling role in the assembly and function of the photosynthetic apparatus. Moreover, severe thermal instability of oxygen yields has been observed in *cpd* mutants [60], suggesting the potential role of *CPD* in photosynthesis. All of these intriguing hypotheses are worthy of further investigation.

### Universality and Characteristics of GmCPDs Compared with AtCPD

The homologous sequences of *CPD* in soybean have not been isolated until the current study. Strong similarities were found between *GmCPDs* and *AtCPD* in many aspects. First, GmCPDs and AtCPD bear high identities in amino acid sequence and structure. Second, *GmCPD1*, *GmCPD2* and *GmCPD4* were most highly expressed in leaves and cotyledons, consistent with the *AtCPD* expression pattern. Most importantly, transformation of *GmCPD* genes into an Arabidopsis CPD-deficient mutant restored the BR biosynthesis pathway and complemented the mutant phenotype with respect to root development, leaf expansion, plant type architecture and flowering regulation, suggesting functional similarity between *GmCPDs* and *AtCPD*.

In addition, *GmCPDs* exhibit some special characteristics in soybean. One is that *GmCPD3* only expresses highly in the young pods of soybean plants. The other is the potential role of *GmCPDs* in soybean flowering regulation. We scanned the entire developmental stage of soybean in a flowering reversion system and found that *GmCPDs* were under photoperiod control. The highest *GmCPD* transcript levels were observed on the 13^th^ day under SD treatment, when the floral meristem initiated. Additionally, *GmCPDs* expressed distinctly in soybean varieties with different photoperiod sensitivities, with insensitive varieties exhibiting higher expression levels especially in the early stages of development. The late flowering phenotype of the *cpd* mutant indicated an essential role of *CPD* in flowering regulation, but the expression patterns of *GmCPDs* in soybean suggested a contributing role of *GmCPDs* in the early stages of flowering development.

Furthermore, all the four *GmCPDs* may perform individual roles and cooperate to regulate flowering. The genomic locations of *GmCPD1* and *GmCPD2* were associated to the QTLs of flower number and the time of the first flower (Fig. 3). Taken into account that *GmCPD2* with the lowest identity of *AtCPD* was not influenced in the transcription level by highly expressed *GmFT2a* while other *GmCPD* homologs decreased in expression (Fig. 11F), *GmCPD1* with the highest identity to *AtCPD* is more likely to play the major role in flowering regulation. Additionally, analysis of SSR markers around *GmCPD3* and *GmCPD4* suggested their association with QTLs of pod maturity and seed quality traits (Fig. 3). This result, taken together with *GmCPD3* specifically expressing in young pods (Fig. 4A), was rather indicated that *GmCPD3* and *GmCPD4* may involve in post-flowering development and fruit ripping. Considering their behavior in flowering regulation, *GmCPD3* and *GmCPD4* are possible to contribute in the whole reproductive stage. Especially *GmCPD4*, bearing similar pattern with *GmCPD1* and *GmCPD2* in flowering regulation, may be the most versatile among this *GmCPD* genes.

### *GmCPDs* Act as Participants in Flowering Regulation

Our study confirmed previous observations that *cpd* mutants exhibit a prolonged vegetative phase and delayed flowering (Fig. 10A, B) [12,34]. This phenotype can be rescued by overexpression of any of the *GmCPDs* we isolated (Fig. 10 A, B). It is therefore clear that *GmCPDs* are associated with flowering. *CPD* has been reported to interact with genes involved in the circadian clock [23,35], the upstream of *FT* in photoperiod pathway. However, in the analysis of *AtFT* expression in wild type, *cpd-91* mutant and mutant with *GmCPDs* transformation, no obvious difference in expression pattern was found (Fig. 10C). In Col-0 Arabidopsis, the expression level of *AtCPD* was higher in vegetative stages and decreased after flowering (Fig. 10D). Therefore, *GmCPDs* may participate in flowering induction. Considering that there was no evidence of changes in flowering time when exogenous BR was applied, thus, *GmCPD* is not the trigger of flowering, acting as a participant rather than a decider.

This hypothesis was illustrated by our analysis of *GmCPD* expression patterns in a flowering reversion system (Fig. 11). The striking observations were that expression of *GmCPDs* is under photoperiod control and is upregulated sharply on the 13^th^ day of SD treatment. The 13^th^ day of SD treatment (13SD) is rather special. In a previous study by our lab, Xiaomei Li *et al* investigated the morphological and anatomical changes that occur during flowering reversion of *Zigongdongdou* [39]. At day 13 under SD condition, the apical meristem began to initiate floral primordia inside the newly formed bracts. Before day 13, the apical meristem retained its vegetative status, and the floral primordia only appeared in the axils of newly formed trifoliolates. The same result also shown by Cunxiang Wu *et al* (Fig. 9) and Hongbo Sun *et al* (Fig. 6) [40,53]; although the lateral floral meristems appeared at SD7, inflorescence differentiation was initiated at the shoot apices at SD13, indicated by the formation of floral meristems and primordia. Logically, these results highlight the potential role of *GmCPDs* in the floral transition of apical meristem.

One possible explanation of the delayed flowering in *cpd* mutants is that floral meristem formation is retarded in the absence of the *CPD* gene, resulting in prolonged flower development manifested as a flowering time delay. How *CPD* participates in floral meristem initiation has not been reported up to now, but the highest level of endogenous BRs and the highest expression of the BR-biosynthesis genes, *DWF4*, *BR6ox1* and *BR6ox2*, have been observed in the apical shoots of Arabidopsis [57]. The effects of BR in cell elongation and cell wall modification is reported to be of vital importance for shoot apical meristem (SAM) function and inflorescence architecture in rice[61]. Further study on the relationship between *CPD* and the shoot apex meristem switch is needed. The new roles of *CPD* in plant development await uncovering.

## Supporting Information

S1 Table Primer sequences used. (DOC)
