# Supplementary material for: Functional Analysis of GmCPDs and Investigation of Their Roles in Flowering
Source: PLoS One. 2015 Mar 3;10(3):e0118476. doi: 10.1371/journal.pone.0118476 (PMC4348418; doi:10.1371/journal.pone.0118476)
Supplement: S1 Table — (DOC) [file pone.0118476.s001.doc]

**Supporting Information**

**S1** Table. Primer sequences used.

| **Role** | **Primer name** | **5' to 3' sequence** |
| --- | --- | --- |
| gene cloning | GmCDP1-F | ATGGCATCTTTCATCATCATACCAC |
| gene cloning | GmCDP1-R | TCATGGCGATTTACTTAGTCTGGAC |
| gene cloning | GmCDP2-F | ATGGCATCTTTCATCTTCACACCTG |
| gene cloning | GmCDP2-R | TCATGGCGATTTACTTAGTTTGGAC |
| gene cloning | GmCDP3-F | ATGGCTTCTTTGCCAGCTTTGCCAA |
| gene cloning | GmCDP3-R | CTAGTCTCTACGCTGCACAATAATT |
| gene cloning | GmCDP4-F | ATGGCTTCTTTGCCAACACTTCTCT |
| gene cloning | GmCDP4-R | CTAATGTCTACGCTGCACAATAATA |
| qRT-PCR, RT-PCR | qGmCPD1-S | GCATCTTTCATCATCATACCACTCC |
| qRT-PCR, RT-PCR | qGmCPD1-A | CAACAAAGGGGAGTCCGAGC |
| qRT-PCR, RT-PCR | qGmCPD2-S | ATGGCATCTTTCATCTTCACACCTG |
| qRT-PCR, RT-PCR | qGmCPD2-A | CGAAGGGGAGCCCGAGTGTAC |
| qRT-PCR, RT-PCR | qGmCPD3-S | ATGGCTTCTTTGCCAGCTTTGC |
| qRT-PCR, RT-PCR | qGmCPD3-A | CGGCGGAGGAAGAGGAGGAG |
| qRT-PCR, RT-PCR | qGmCPD4-S | ATGGCTTCTTTGCCAACACTTCTCT |
| qRT-PCR, RT-PCR | qGmCPD4-A | GAACACGCGGCGGAGGTATAAG |
| qRT-PCR, RT-PCR | AtACTIN2-S | ACTCTCCCGCTATGTATGTCGC |
| qRT-PCR, RT-PCR | AtACTIN2-A | AGAAACCCTCGTAGATTGGCAC |
| qRT-PCR | GmG6PDH-S | GTCTGTTATCCGCCTACAGCCT |
| qRT-PCR | GmG6PDH-A | ACTCCTTGATACCGTTGTCCAT |
| qRT-PCR | AtCPD-S | CCTTGGAGATGGCAGCAA |
| qRT-PCR | AtCPD-A | GTAACCGGGACATAGCCTTG |
| qRT-PCR | AtFT-S | CTTGGCAGGCAAACAGTGTATGCAC |
| qRT-PCR | AtFT-A | GCCACTCTCCCTCTGACAATTGTAGA |
| qRT-PCR | GmFT2a-S | GCTGACATCTCTGTTATTGTAGGTA |
| qRT-PCR | GmFT2a-A | TAATTCATAACAAAGCAAACGAGTA |
